# 5G-BSS: 5G-Based Universal Blockchain Smart Sensors

**DOI:** 10.3390/s22124607

**Published:** 2022-06-18

**Authors:** Zhengyi Yao, Liang Tan, Kun She

**Affiliations:** 1 College of Computer Science, Sichuan Normal University, Chengdu 610101, China; 20201301016@stu.sicnu.edu.cn; 2 Institute of Computer Science, Chinese Academy of Sciences, Beijing 100190, China; 3 College of Information and Software Engineering, University of Electronic Science and Technology of China, Chengdu 610056, China; kun@uestc.edu.cn

**Keywords:** smart sensor, blockchain, 5G communication, distributed storage system

## Abstract

A smart sensor is a sensor with information processing functions. It is the product of the combination of sensor integration and a microprocessor. It has the characteristics of intelligence, networking and high precision. It has been widely used in aerospace, aviation, intelligent transportation, industrial control and medical and health care. However, in some specific application scenarios with high data security requirements and low transmission delay, such as environmental detection, transportation, etc., smart sensors have three obvious shortcomings. First, the data transmission delay is high. Second, the confidentiality and integrity of the data transmission process cannot be effectively guaranteed. Third, centralized data storage is easily leaked and tampered with by malicious users and semi-trusted administrators. Therefore, a 5G-based blockchain smart sensor 5G-BSS was designed. 5G-BSS has three innovation points. First, the 5G communication module enables the smart sensor 5G-BSS. The 5G communication module is integrated into the smart sensor 5G-BSS to reduce the delay of data transmission and improve the speed and reliability of data transmission. Second, cryptographic algorithms enable the smart sensor 5G-BSS. The data encryption module of the smart sensor 5G-BSS improves the confidentiality and integrity of the data transmission process. Third, blockchain empowers the smart sensor 5G-BSS. The blockchain client is integrated into the smart sensor 5G-BSS to ensure the centralized storage of data and prevent data leakage and tampering by semi-trusted administrators. The operation process of the hardware and software architecture is described in detail and tested on the Fisco-Bcos. The experimental results show that 5G-BSS not only has fast data transmission speed but also can effectively guarantee the integrity, confidentiality and availability of data. 5G-BSS is suitable for application scenarios with high requirements for data security and data transmission, such as environmental monitoring, intelligent transportation, autonomous driving, etc.

## 1. Introduction

Smart sensors are the result of the integration of sensitive components combined with microprocessors. Smart sensors integrate sensor detection information functions and microprocessor data processing functions. Compared with general sensors, smart sensors have the following advantages: they can achieve high-precision information acquisition and fast data processing; they are low cost; they have the ability to transmit information for data interaction; they have diversified functions; they are intelligent and have low power consumption, etc. Therefore, smart sensors have gradually been used in various fields, such as aerospace, aviation, national defense, science and technology, as well as industrial and agricultural production [1,2,3,4,5]. For example, there is an instance in the field of robotics [6] where smart sensors enable robots to have human-like functions of the five senses and the brain to sense various phenomena and perform various actions. In industrial production [7,8,9], certain product quality indicators (e.g., viscosity, hardness, surface finish, composition, color and flavor) cannot be quickly and directly measured and controlled online using conventional sensors. However, some physical quantities in the production process (such as temperature, pressure, flow, etc.) are functions of product quality indicators that can be directly measured using smart sensors and calculated using mathematical models based on neural network or expert system technology, which can infer the quality of the product. In the field of medicine [10], smart sensors were integrated into glucose watches using composite pH-sensitive sensors, and a new glucose-sensitive potentiometric biosensor was developed using composite pH-sensitive sensors, characterized by EIS and CV measurements. In particular, with the rapid development of semiconductor technology in recent years, several well-known foreign companies and institutions of higher education are making great efforts to develop the integration of smart sensors. Some of the country’s leading universities and research institutes and companies are also actively following suit, with smart sensors rapidly becoming a hotspot for research in the field of the Internet of Things (IoT) and automatic control.

In recent years, 5G communication technology and blockchain technology have been developing rapidly. The Fifth Generation Mobile Communication Technology (5G) is a new generation of broadband mobile communication technology with high bandwidth, low latency and support for large connections, and is a network infrastructure for the interconnection of people, machines and things. According to ITU-R, 5G communication scenarios can fall into three categories: enhanced mobile broadband (eMBB), ultra-high reliability low latency communication (uRLLC) and massive machine-type communication (mMTC) [11,12]. Among them [13], enhanced mobile broadband (eMBB) supports stable connections with very high peak data rates, as well as medium rates for cell-edge users, mainly in response to the explosive growth of mobile internet traffic, providing a more extreme application experience for mobile internet users. Massive machine-type communication (mMTC) supports a large number of Internet of Things (IoT) devices that are only occasionally active and send small data payloads, mainly in application scenarios that target sensing and data collection, such as smart cities, smart homes and environmental monitoring, etc. Ultra-high reliability low latency communication (uRLLC) supports low latency transmission with very high reliability from a limited number of terminals for applications where latency and reliability requirements are particularly stringent, such as industrial control, telemedicine and autonomous driving, etc. The concept of bitcoin was first proposed in 2008 and implemented by Satoshi Nakamoto in 2009 [14]. Since then, it has seen tremendous growth in the capital markets, reaching US10 billion in 2016. Bitcoin’s underlying technology, blockchain, is a decentralized database, assets and related transactions ledger [15]. Enabling data sharing among nodes within the network, blockchain has its outstanding features such as decentralization, transparency, robustness, auditability and security [16,17], which in turn are widely used in applications, such as the Internet of Things [17], healthcare [18], Industry 4.0 [19], logistics and transportation [20] and financial services [21], etc. A USBKey is a USB hardware device that provides secure data storage and password-secure computing services. The device has a built-in microcontroller or smart chip and can only be accessed through a specific interface. Due to its ultra-high security features [22], the USBKey is widely used in e-commerce [23], file systems [24], software security [25], etc.

However, due to the wide application of smart sensors, we found that in some special application scenarios with high data security requirements and low transmission latency, such as environmental inspection and transportation, etc., there are three obvious disadvantages: firstly, data transfer latency is high; secondly, the confidentiality and integrity of the data transmission process are not effectively guaranteed; thirdly, the centralized data storage is vulnerable to leakage and tampering by malicious users and semi-trusted administrators. For example, in the field of the Internet of Vehicles (IoV), smart sensors have been widely used in driverless cars, where smart sensors collect information about the surrounding roads and the unmanned status of the car, and real-time access to this information data plays an important role in the process of auto-driving, while high latency and low bandwidth data transmission are one of the key factors hindering the realization of highly reliable driverless cars. Another example is in the field of environmental detection, where sensors collect data on physical variables in the environment, then transmit to multi-level edge devices for collation and collection and finally transmit to control back end systems to apply data. This transmission process is complex and bloated for a long time and is susceptible to security risks, such as data eavesdropping, signal interference and communication artefacts in the transmission process. Currently, most of the data collected by sensors eventually choose to be stored in the cloud center, which is a centralized storage method. Cloud-centric storage is semi-trustworthy and may be subjected to security risks, such as hardware failure, software failure, human failure and natural disasters, etc., resulting in data tampering or even data loss.

Therefore, this paper proposes a 5G-based blockchain smart sensor solution using 5G communication technology and blockchain technology to address the three drawbacks above. A new smart sensor has been designed by us, namely a blockchain smart sensor based on 5G communication, called 5G-BSS. An encryption module and blockchain client module were added to 5G-BSS, using the USBKey peripheral to provide the encryption secret key and save the relevant parameters of the blockchain client, and the Huawei Baron MH5000-31 5G communication module was selected as the network adapter for 5G-BSS. The main research contents and contributions of this paper are as follows:

(1) The 5G communication module enables the smart sensor 5G-BSS. Integrating the 5G communication module into the smart sensor 5G-BSS, making full use of the low latency, high reliability and high speed of 5G technology, can quickly upload the data collected by the sensor module and respond in a timely manner, effectively solving the data transmission delay, high, slow and other issues.
(2) Cryptographic algorithms enable the smart sensor 5G-BSS. The encryption module and the USBKey are integrated into the smart sensor 5G-BSS, so that the 5G-BSS applies cryptographic algorithms to ensure the confidentiality and security of data transmission when uploading the collected data.
(3) The blockchain empowers the smart sensor 5G-BSS. By integrating the blockchain client into the smart sensor 5G-BSS, the 5G-BSS can upload the collected data to the blockchain, making full use of the features of blockchain decentralization, tamper proof, traceability, etc., to effectively solve the problem. It solves the semi-trusted problem of using centralized storage of data and administrators.

In summary, 5G-BSS can better solve the above three deficiencies and can be applied to scenarios with high real-time and safety requirements, such as autonomous driving, intelligent transportation, intelligent logistics, environmental monitoring, etc.

The rest of the paper is organized as follows. Section 2 of the paper briefly describes the research results of scholars on smart sensors. Section 3 describes the design scheme of the 5G-BSS in detail. Section 4 analyzes the features and security of the 5G-BSS. Section 5 presents the prototype experiments of the 5G-BSS, completing the functional verification and performance testing of the 5G-BSS. Section 6 summarizes the whole thesis and provides an outlook on the future development of smart sensors.

## 2. Related Work

In recent years, many scholars have made many achievements in the research of smart sensors. In the following, we will present the current status of research on smart sensors from three aspects: smart sensor performance indicators, smart sensors combined with blockchain and smart sensors combined with 5G.

In terms of smart sensor performance indicators, current research work of smart sensors focuses on high accuracy, integration, miniaturization, high reliability, networking and wireless energy [1,26,27]. Lamba et al. [28] used the prepared porous ZnO-SnO_2_ nanosheets to fabricate a robust chemically smart sensor, which exhibited extremely high sensitivity in detecting 4-nitrophenol in aqueous media. Siangdee et al. [29] developed a cellulose-based material smart sensor for the detection of 2,4,6-trinitrotoluene (TNT), using a potassium hydroxide/ethanol reagent adsorbed on a cellulose swab. The smart sensor was used to detect TNT samples, such as soil, non-porous and porous surfaces, with high accuracy and reliable performance for detection of TNT. Kim et al. [30] developed a multifunctional contact lens smart sensor that was developed on an actual ocular contact lens, which can be used to monitor glucose in tears, as well as monitor intraocular pressure using the resistance and capacitance of the electronic device. Adamo et al. [31] proposed a smart sensor network system based on the ISO/IEC/IEEE 21451 standard, which is used for in situ and continuous spatiotemporal monitoring of surface waters, especially seawater, and the system provides effective support for strategic decision making on key environmental issues. Miller et al. [32] studied the development of a solar harvesting system to power the Imote2 WSS platform and improve the long-term autonomy of a wireless smart sensor network (WSSN). The papers [28,29] investigate new sensitive materials and explore novel sensing methods, arraying and compounding sensitive elements to achieve high accuracy and reliability of smart sensors. The research in the thesis [30] is in line with the development of integration and miniaturization of smart sensors. Paper [31] embodies research on smart sensor networking and paper [32] investigates research on wireless energy.

In terms of combining smart sensors with blockchain, Lukman [33] mentions a vehicle safety tracking system for remote monitoring of oil shipments using the cloud. Firstly, where a GPS receiver is used to obtain the vehicle position and ultrasonic sensors to monitor the tanker fluid stages at intervals, the database is hashed using blockchain technology, using SHA-1 algorithms. Finally, all the data is saved to the database so that it can be linked to subsequent records. Voicu et al. [34] proposed a scheme using IoT smart sensor nodes to monitor environmental parameters, designing smart sensors for collecting barometric data, using the ESP8266 WiFi module for data communication and storing the collected data in a temporal database (InfluxDB). Roman et al. [35] proposed an IoT system solution, which collected, sent, stored and published relevant data in Raspberry Pi as a smart sensor, and stored the data to BigChainDB. Papers [33,34,35] are all sensor applications of incomplete blockchains. Paper [33] is an SHA-1 algorithm that is utilized to link data to subsequent data for storage. Paper [34] uses a temporal database to store the data. The system solution in the paper [35] only uses BigChainDB and Distributed Ledger Technology (DLT) being used as a blockchain-like database to store data. Some of the features of blockchain are utilized in these schemes, but not a fully decentralized storage sensor data storage application.

In terms of combining smart sensors with 5G, Ojanperä et al. [36] present a new 5G road safety service use case and framework in which vehicle sensors and roadside infrastructure sensors collect and acquire road data; data communication is achieved through 5G communication technology and data processing can be partially or fully placed on the MEC server. Divya and Chinnaiyan [37] pointed out the use of 5G technology and miniature sensors for the detection of some vital features and the transmission of data at high speed, high capacity, low latency and low cost. The paper also describes a small earbud smart sensor designed through 5G technology and smart sensor technology. Jia et al. [38] designed a wireless I/O (input or output) system. The system utilizes a 5G local area network (5G-LAN) for the transmission of I/O signals, and remote modules of the system include sensors and actuators for data acquisition and output. Papers [36,37,38] all use 5G communication technology to complete the transmission of sensor data, because 5G communication technology has the advantages of high bandwidth, low latency and high security. It is not difficult to realize that the combination of 5G communication technology and smart sensors is an irreversible trend.

Furthermore, an extensive search of the literature revealed very few relevant studies that directly combine smart sensors with blockchain and 5G. Only a few academic studies of platforms or frameworks exist that jointly use blockchain technology, 5G communication technology and smart sensor technology. Han, Park and Jeong [39] proposed a real-time air pollution index monitoring platform using a 5G wireless network and blockchain technology. The platform uses IoT sensors to collect data in real-time, transmits the data via 5G wireless communication technology and uses blockchain technology to encrypt in the data retention layer, finally transmitting data to the cloud. Reebadiya et al. [40] proposed a framework for security and intellisense, which uses blockchain technology and 5G communication technology. The framework is used to address the sensing and tracking technologies and data theft that are being faced in self-driving vehicles. Blockchain technology protects AV systems from attacks and security countermeasures, uses 5G networks to transmit data collected by onboard sensors and deploys artificial intelligence algorithms on edge servers.

In summary, the blockchain smart sensor based on 5G communication proposed in this paper has not yet been studied by scholars, and the technology of 5G communication and blockchain has only been used in some IoT platforms and frameworks. The comparative results of their work are shown in Table 1. However, the smart sensor designed in this paper is also very different from the above-mentioned platforms and frameworks and 5G-BSS has two advantages. Firstly, in 5G-BSS, the data collected by the sensor is directly transmitted to the blockchain storage system for storage via 5G wireless communication technology without the need to relay the data through edge computing devices, reducing the complexity of the data transmission path and ensuring a more secure data transmission based on the high reliability of 5G communication technology. Secondly, data processing occurs with data encryption and data hashing in 5G-BSS, rather than using blockchain security technology for processing and then still using semi-trusted cloud storage for continued use. 5G-BSS ensures more data integrity, availability and confidentiality. Therefore, the 5G-BSS designed in this paper is more suitable for high security and low latency scenarios, such as environmental monitoring, intelligent transportation, industrial control, etc.

## 3. Overall Design of 5G-BSS

### 3.1. Hardware System Design

#### 3.1.1. Hardware Architecture

**Figure 1:**
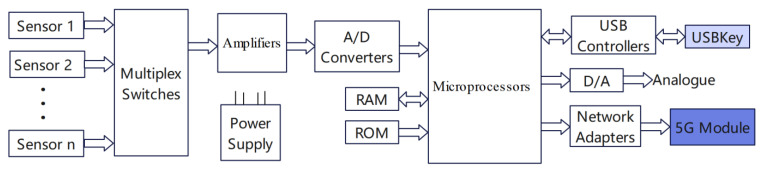
Diagram of 5G-BSS structure.

Smart sensors are mainly composed of conventional sensors, multiplex switches, amplifiers, A/D converters, microprocessors (or microcomputers) and related circuits. Traditional sensors will be measured by the physical quantities into the corresponding electrical signals, which will be sent to the signal conditioning circuit, filtering, amplification, analogue-to-digital conversion and sent to the microprocessor (or microcomputer). A microprocessor (or microcomputer) is the core of smart sensors; it can not only calculate the sensor measurement data, storage, and data processing but also through the feedback loop to adjust the sensor. As the microprocessor (or microcomputer) effectively performs a variety of software functions, it can complete tasks that are difficult to complete with hardware. As a result, the manufacturing difficulty of the smart sensor is greatly reduced, the performance of the smart sensor is improved and the cost is reduced. The 5G-BSS structure designed in this paper is shown in Figure 1.

#### 3.1.2. Hardware Selection

Open-source hardware is a hardware artefact that can be manufactured, modified, distributed and used by anyone. Commonly used open-source hardware are Arduino, Raspberry Pi (RPi), vvBoard and Mirco: bit, etc. A comparison of these open-source hardware parameters is shown in Table 2. The choice of open-source hardware in the hardware design of 5G-BSS will enable the rapid construction of a hardware prototype of the blockchain smart sensor. Three main software tasks need to be run by 5G-BSS. Task 1: to receive data on physical variables from the surrounding environment from an acquisition module consisting of inductive sensors. Task 2: processing of incoming physical variable data, including integrity processing and confidentiality processing. Task 3: using the network of deployed blockchain nodes and the already installed blockchain client, call the smart contract interface for data transfer and quickly upload the processed data to the blockchain via 5G network communication technology. For the analysis of these three running tasks and the comparison with the results in Table 2, we found that the Raspberry Pi 4B—a RAM-based microcomputer motherboard with the advantages of high performance, large memory and rich interface—can perform these three tasks well. Therefore, the Raspberry Pi 4B is chosen as the hardware platform for the blockchain smart sensor in this paper.

The network adapter module for smart sensors has been selected for 5G communication, which will enable fast transmission of data collected by the sensors. At present, 5G communication mobile phones have gradually become popular in China, Beijing, Shanghai, Shenzhen and Chengdu, and 17 other cities have achieved the use of 5G mobile phones by civilians. The current mainstream 5G mobile chips are: Kirin 990 with integrated 5G baseband, Qualcomm Snapdragon 855/865 with external X55 baseband, Qualcomm Snapdragon 888 with integrated X60 baseband, Tiangui 1000 with integrated Helio M70, etc. The performance goals of 5G communications are to increase data rates, reduce latency, save energy, reduce costs, increase system capacity and support large-scale device connectivity. With the rapid advancement of 5G infrastructure and the continued commercialization of 5G communications, 5G base stations are dense, providing realistic conditions for the application of 5G communications to the Internet of Things (IoT) and the Industrial Internet of Things (IIoT). 5G modules are based on 5G chips, with external circuitry added according to actual demand and then packaged. There are only two types of mainstream 5G industrial communication modules currently available on the market. One is the Baron MH5000 module with integrated Huawei 5G baseband and the other is the SIM8200EA-M2 module based on the Qualcomm Snapdragon X55 baseband. A partial comparison of their parameters is shown in Table 3. By comparing the parameters of the two 5G communication modules, it can be seen that Huawei’s Baron 5000 module has three major advantages. First, it offers high data rate, low latency, energy saving, low cost and support for large-scale device connectivity. Second, Baron 5000’s 5G module supports Huawei’s self-developed interfaces, including Modem, PCUI, Diag, GPS and GPS Control, etc. Third, it can perfectly dock with Ubuntu/Linux system to quickly build a communication environment. Therefore, the Huawei MH5000-31 5G module is chosen as the network adapter for the blockchain smart sensor.

In this solution, it is necessary to use the USBKey to provide the smart sensor secret key and save the relevant parameters of the blockchain client and the address of the smart contract; therefore, the USBKey needs to complete three tasks. Firstly, the smart sensor secret key includes a symmetric encryption secret key and an asymmetric secret key, which is calculated by the USBKey cryptographic computing service and stored in the USBKey. Secondly, after the client is initialized, the relevant parameters of the blockchain client will be generated. These parameters are sensitive data that need to be stored, including node-related digital certificates, etc., which are written into the USBKey storage area by calling the USBKey-related interface. Thirdly, the user of the smart sensor saves the smart contract address in the USBKey through the computer calling the write interface, and the USBKey provides the address to the 5G-BSS. Currently, domestic and international information security technology companies offer their USBKey product solutions, which differ somewhat in terms of data security storage and password security computing services. This paper analyzes and compares the parameters of the mainstream USBKey products, the results of which are shown in Table 4, combining the three tasks that the USBKey needs to complete and the parameter comparison results of USBKey products. The LinguoTech USBKey can provide an SM2-256 asymmetric key and securely store sensitive data, secret key generation algorithm, true random number generation algorithm and rich password service interface, etc. Therefore, we choose the LinguoTech USBKey as the USBKey peripheral to serve the smart sensor.

Therefore, the hardware options for 5G-BSS include the Raspberry Pi 4B, the Huawei MH5000-31 5G industrial module and the LinguoTech USBKey. The network adapter of 5G-BSS uses the multifunction interface of the Huawei Barong 5G industrial module, which can better connect with the Raspberry Pi 4B. In this way, a Linux-based network communication system can be quickly configured and constructed. 5G-BSS uses the Huawei 5G communication module MH5000-31 based on the Baron series 5G chips to realize data transmission. The LingoTech USBKey provides secret keys, password service calculation, data security storage, etc.

#### 3.1.3. Hardware Interface Design

**Figure 2:**
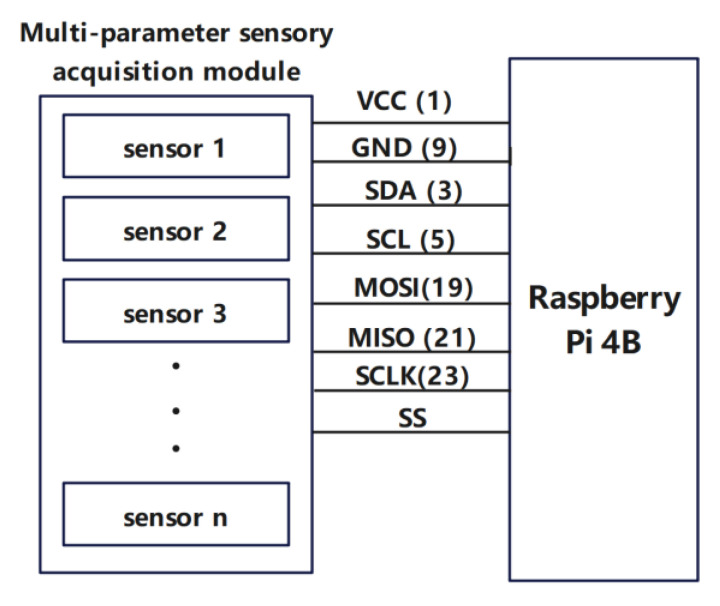
Hardware design of the interface between the Raspberry Pi 4B and the multiparameter sensing acquisition module.

**Figure 3:**
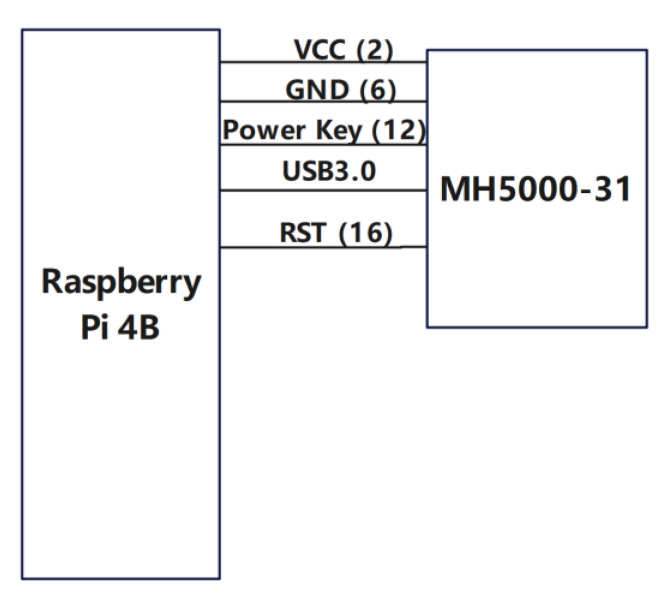
Hardware design of the interface between Raspberry Pi 4B and the 5G communication module.

For the hardware interface design of 5G-BSS, the hardware interface diagram of the Raspberry Pi 4B and the multi-parameter sensor acquisition module is shown in Figure 2. The hardware interface diagram of the Raspberry Pi 4B and the Huawei Balong MH5000-31 5G industrial module is shown in Figure 3. Note that the value in parentheses is the physical pin BOARD code of the Raspberry Pi 4B.

In Figure 2, the multiparameter sensing module is connected to the hardware interface of the Raspberry Pi 4B microprocessor. The Raspberry Pi 4B supplies power to each sensing module in the multiparameter sensing module via VCC (Volt Current Condenser) and GND (Ground) with a current limit of 5 A at 3.3 V. The Raspberry Pi 4B microprocessor uses the IIC (Inter-Integrated Circuit) bus and SPI (Serial Peripheral Interface) bus to communicate with the multiparameter sensing acquisition modules. The Raspberry Pi 4B microcontroller acts as the IIC host and multiple sensors collecting data act as IIC slaves, with SCL acting as the clock line for IIC bus communication and SDA acting as the data line for IIC bus communication. The master-slave communication mode is used in the SPI bus, with the Raspberry Pi 4B microcontroller acting as the master device and multiple collecting data sensors all acting as slaves, where Serial Clock (SCLK) is the clock signal generated by the master device, Master out/Slave in (MOSI) is the master device outputting the slave device input, Master in/Slave out (MISO) is the master device input and slave output and Slave Select (SS) is the master device’s chip select signal to control the slave devices.

In Figure 3, the Raspberry Pi 4B is connected to the hardware interface of the Huawei MH5000-31 module. The supply to the Huawei MH5000-31 5G industrial module is via VCC (Volt Current Condenser, the supply voltage of the circuit) and GND (Ground), with a voltage of 5 V limited to 6 A. The Power Key (physical pin number 12, GPIO18) is the power switch signal of the Baron MH5000-31 5G module. When GPIO18 outputs a high level continuously for 1.5 s, the power key of the Huawei MH5000-31 module is on and the Huawei MH5000-31 module starts to boot up and starts working. RST (physical pin number 16, GPIO23) is the hardware reset signal pin of the Huawei MH5000-31 module, when GPIO23 is low the Huawei MH5000-31 module is reset. The Raspberry Pi 4B sends and receives data with the Huawei MH5000-31 module through the USB 3.0 interface, thereby realizing information transmission. The controller (Raspberry Pi 4B) sends AT commands through the USB3.0 interface to read the status of the Huawei MH5000-31 5G module.

#### 3.1.4. Huawei MH5000-31 5G Module Development Based on Raspberry Pi 4B

**Figure 4:**
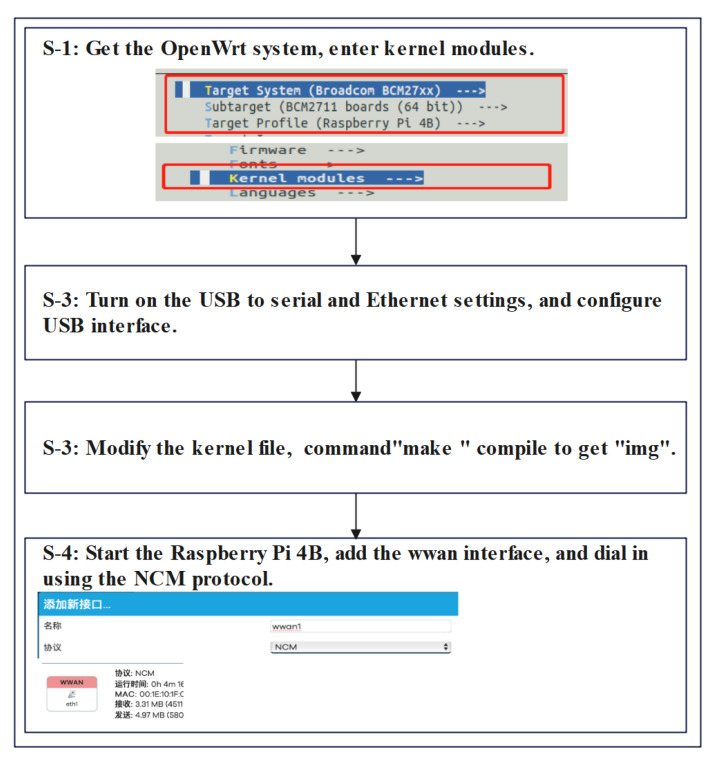
Schematic diagram of the Huawei MH5000-31 5G module development steps based on Raspberry Pi 4B.

From the previous hardware selection and hardware interface design, it can be seen that the hardware platform of 5G-BSS mainly consists of Raspberry Pi 4B, the Huawei MH5000-31 5G module, the USBKey and the multiparameter acquisition module. An excellent and reliable 5G-BSS hardware platform is the basis for the 5G-BSS software system design and is necessary for the stable and efficient operation of 5G-BSS. The hardware platform environment of 5G-BSS includes the following: the connection of Raspberry Pi 4B with the Huawei MH5000-31 5G module, the USBKey and the multiparameter acquisition module. Among them, the Raspberry Pi 4B and the USBKey connection is only required to secure the USB driver; Raspberry Pi 4B and the multiparameter acquisition module only need to install SPI, IIC and other communication bus drivers. This subsection does not go into detail. Therefore, this subsection focuses on the development steps of the Huawei MH5000-31 5G module based on the Raspberry Pi 4B, which is shown in Figure 4.

OpenWrt [41] is a highly modular, highly automated embedded Linux distribution with a simple compilation and installation process and works perfectly with the Raspberry Pi 4B. Therefore, the OpenWrt system is selected for the Raspberry Pi 4B hardware platform. From Figure 4, we can see that there are four main steps in the development of the Huawei MH5000-31 5G module based on Raspberry Pi 4B as follows.

S-1: obtain OpenWrt 19.07 from the official OpenWrt website [41] and execute the commands “. /script/feeds update -a” and “. /script/feeds install -a”, go to “Target System (Broadcom BCM27xx)” and select “Kernel modules”.

S-2: Open the USB to the serial port, because the AT command [42] of the 5G module needs to be sent and received via “/dev/ttyUSB”. Install the USB to the Ethernet driver. Install mwan3, because the Raspberry Pi 4B has multiple USB 3.0 ports for load balancing and bandwidth aggregation at work. Turn on the 5G module related configuration switch in the kernel via “USB support”.

S-3: Modify the Linux kernel file according to the Huawei module information; the kernel files are “driver/usb/serial/option.c” and “driver/usb/serial/usb_wwn.c”. Then, use the “make” command to compile and generate “img” in the “bin/targets/bcm27xx/bcm2711” directory after successful compilation. Finally, write the generated “img” to the SD card.

S-4: Insert the SD card into the Raspberry Pi 4B and turn it on. Raspberry Pi 4B is physically connected to the Huawei MH5000-31 5G module via a USB3.0 interface. Use “ifconfig -a” command to check eth1 NIC, then, add interface “wwan” and use NCM to dial up the internet. Finally, to test the network, the Raspberry Pi 4B can transmit data through the Huawei MH5000-31 5G module.

### 3.2. Software System Design

#### 3.2.1. General Structure of the Software System

**Figure 5:**
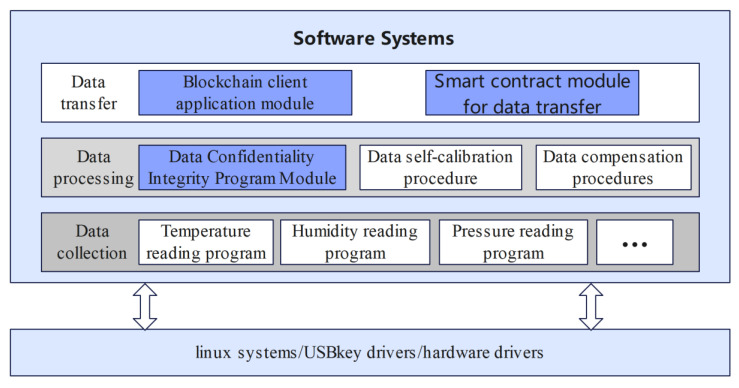
Software overall architecture block diagram.

As can be seen from Figure 5, the software system of 5G-BSS designed in this paper contains three layers, namely: the data collection layer, the data processing layer and the data transmission layer.

- The main function of the data collection layer is to collect the target data. Since the hardware selected for 5G-BSS is Raspberry Pi 4B, it has good hardware interface scalability. The multiparameter sensing acquisition module of 5G-BSS can support the simultaneous access of multiple traditional sensors, so it can collect various data at the same time, e.g., ambient temperature, humidity, pressure, etc.
- The main function of the data processing layer is to process the various data collected by the sensors. Conventional sensors that collect different types of data have corresponding processing modules, such as data self-checking modules, data compensation modules, etc., which will not be repeated in this article. In order to ensure the confidentiality and integrity of sensor data, this paper focuses on designing a data integrity protection module and a data encryption module.
- The main function of the data transmission layer is to upload data to the blockchain. In order to upload the data to the blockchain, a blockchain client module and a data transmission smart contract module are designed in the data transmission layer. The blockchain selected in this paper is Fisco-Bcos; Fisco-Bcos is a federated chain. The reasons for choosing Fisco-Bcos are that: (1) Fisco-Bcos has better performance, security, operability, ease of use, scalability, etc.; (2) the data is uploaded through the JDK provided by Fisco-Bcos and the data transmission smart contract module developed by us. The amount of code is small and the configuration is simple and convenient. It is especially suitable for the hardware platform chosen in this paper.

In the following, we will focus on the data confidentiality and integrity program in the data processing module, the blockchain client initialization process and the data transfer smart contract program module.

#### 3.2.2. The Initialization of the Blockchain Client Program Module

In order to make 5G-BSS nodes become legitimate users of the blockchain, it must first install and initialize the blockchain client program. The core task is to install the blockchain client program on the 5G-BSS node, making it a light node on the blockchain and registering a transaction address on the blockchain. First, the blockchain smart sensor requests the secret key from the USBKey, which consists of the asymmetric secret-key pair KSN and the symmetric secret-key Key. Then, the blockchain smart sensor uploads the *K_pubSN_* to the blockchain, which uses the public key *K_pubSN_* to generate into a transaction address *Addr* for the smart sensor node and returns *Addr* to the smart sensor node. Finally, the blockchain smart sensor calls the interface of the USBKey to store the parameters related to the initialization process of the blockchain client into the USBKey. To accurately describe this process, we first define some of the important symbols and functions used in this section, as shown in Table 5.

**Figure 6:**
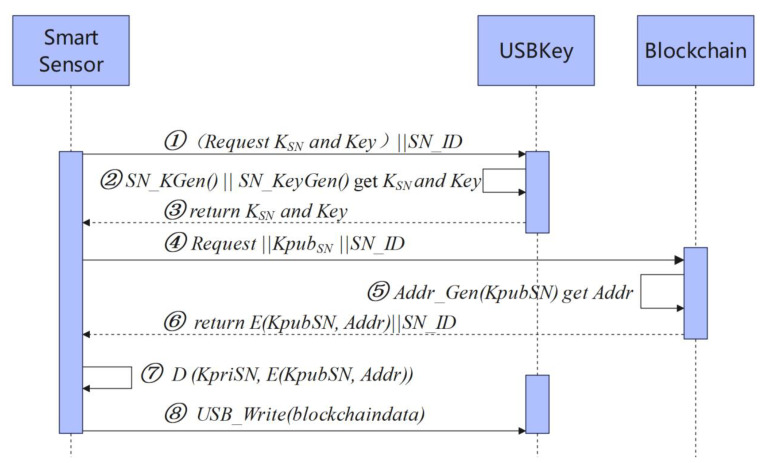
Blockchain client program module initialization process.

The registration process for a smart sensor node is shown in Figure 6 and consists of five steps.

① SN -> USBKey: (*Request K_SN_* and *Key)*||*SN_ID*. The smart sensor requests the USBKey for the asymmetric secret-key pair *K_SN_* and the symmetric secret-key *Key* and sends the smart sensor number *SN_ID*.

② USBKey: *SN_KGen()*||*SN_KeyGen()* retrieve *K_SN_* and *Key*. The interface *SN_KGen()* is called to generate the asymmetric secret-key pair *K_SN_* for the smart sensor, including the public key *K_pubSN_* and the private key *K_priSN_*, and the interface *SN_KeyGen()* is called to generate the symmetric secret-key *Key* for the smart sensor.

③ USBKey -> SN: *K_SN_* and *Key*. USBKey sends asymmetric secret-key pair *K_SN_* and symmetric secret-key *Key* to the smart sensor.

④ SN -> Blockchain: *Request*||*K_pubSN_*||*SN_ID*. The smart sensor sends registration requests *Request*, *K_pubSN_* and *SN_ID* to blockchain.

⑤ Blockchain: *Addr_Gen(K_pubSN_)*. The blockchain calls interface *Addr_Gen (K_pubSN_)* to generate the blockchain account address *Addr* for the smart sensor.

⑥ Blockchain -> SN: *E(K_pubSN_, Addr)*||*SN_ID*. The blockchain returns *SN_ID* the ciphertext of the smart sensor blockchain account address *Addr*.

⑦ SN: *D (K_priSN_, E(K_pubSN_, Addr))*.The smart sensor decrypts the ciphertext *D(K_pubSN_, Addr)* to retrieve the account address *Addr*.

⑧ SN -> USBKey: USB_Write(*blockchiandata*). The smart sensor calls the USBKey’s write interface to write *blockchaindata* about parameters related to the blockchain client initialization process, *blockchaindata* contains blockchain client account address *Addr*, node digital certificate, smart contract address, etc.

#### 3.2.3. Data Integrity and Confidentiality Program Module

The Fisco-Bcos blockchain does not support confidentiality, hence some encryption mechanisms were integrated into 5G-BSS. This subsection focuses on a brief introduction to the software design of the data integrity and confidentiality program module in 5G-BSS. In 5G-BSS, the data is hashed to ensure integrity and the data is kept confidential using cryptographic operations. In this regard, the hash value obtained from the hashing operation is used to verify data integrity and the hash function is chosen from the SM3 hash algorithm [43]. The encryption operation uses the symmetric encryption algorithm to encrypt the sensor data, the encryption secret key is the USBKey peripheral into the smart sensor symmetric secret key *Key*. The encryption function is the SM4 symmetric encryption algorithm [44]. The encryption operation can have good confidentiality for data processing. It should be noted that the USBKey peripheral is only owned by the smart sensor purchaser. Therefore, this paper designed the data processing functions of the smart sensor data integrity and confidentiality program, as shown in Algorithm 1.
**Algorithm 1:** Smart sensor data integrity and confidentiality algorithms**Input:** *SensorData, Key***Output:** *CipherData***Begin**1. Retrieves the current timestamp *timestamp* and *SN_ID*2. *ProData*=*SN_ID*||*SensorData*||*timestamp*;3. *DataHash*=*SM3(ProData)*;4. *CipherData* =*SM4(Key,ProData||DataHash)*;5. return *CipherData*;**End**

The parameters of Algorithm 1: *SensorData* is the sensor data to be stored, *Key* is the symmetric secret key to import the smart sensor node. In Algorithm 1, *SN_ID* is the unique number of the smart sensor, t*imestamp* is the current timestamp and || indicates data stitching. The first step is to get the current timestamp *timestamp* and the serial number *SN_ID* of the smart sensor device. The second step stitches together *SN_ID*, sensor data *SensorData* and the current timestamp *timestamp* to form *ProData*. The third step calls the *SM3()* hash function to calculate. The fourth step calls the *SM4()* symmetric encryption function to encrypt the data using *Key* as the secret key, where the plaintext is the stitching of *ProData* with *DataHash* and the output ciphertext is *CipherData*. The fifth step returns the *CipherData*.

#### 3.2.4. Smart Contract Module for Data Transfer

The most important thing in a blockchain smart sensor system is to upload data of environmental variables to the blockchain for preservation; the data is processed for integrity and confidentiality. Before designing a smart contract, the function ISave is first introduced. *ISave()* is a blockchain storage interface with input parameters of address, private key, stored content and timestamp, and that with success returning as transaction ID and failure returning 0. The interface that implements the data upload to the blockchain save function is the smart contract *UpData*(*Data*, *K_priSN_*, *Addr*). In the smart contract UpData, *Data* represents the data to be uploaded, *K_priSN_* is the private key of the smart sensor node and *Addr* is the address of the smart sensor blockchain node, which returns “*tranID*” on success and “*0*” on failure, as shown in Smart Contract 1.
**Smart Contract 1:** Data Upload Smart Contracts**Input:***Data*, *K_priSN_*, *Addr***Output:** *tranID***Begin**1. if(*Data*==*null* || *K_priSN_*==*null* || *Addr*==*null*)2.  *tranID* = 0;3. else4.  Retrieves the current timestamp *timestamp*;5.  *tranID* = *ISave(Addr*, *K_priSN_*, *Data*, *timestamp)*;6. return *tranID*;**End**

The smart contract *UpData(Data*, *K_priSN_*, *Addr)* in Smart Contract 1: The first step determines the correctness and reasonableness of the input data. The second step obtains the current timestamp *timestamp*. The third step calls the blockchain storage interface *ISave()* to issue a data preservation transaction. The fourth step returns the *TranID*.

### 3.3. Smart Sensor System Operation Process Design

**Figure 7:**
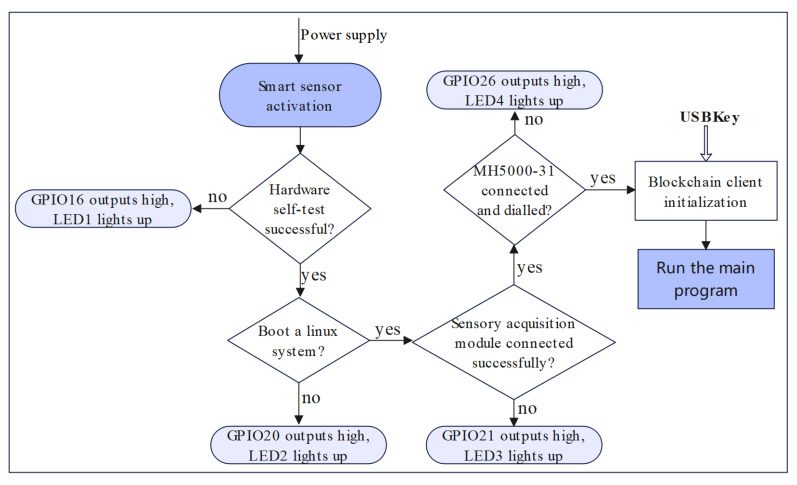
Flowchart of the operation of 5G-BSS.

After completing the hardware selection, hardware system design and software system design of 5G-BSS, this subsection will carry out the design of the running process of the blockchain smart sensor system. The smart blockchain sensor system based on 5G communication is run on a Linux system, and the running process of the smart sensor is mainly divided into seven steps to start and finally run stably and efficiently, collecting data, processing data and uploading data. The workflow diagram of the smart sensor is shown in Figure 7.

S-1: Apply normal power to the smart sensor and then power it on.

S-2: The hardware of the core module (Raspberry Pi 4B) is tested, if there is a hardware failure of the core module, GPIO16 outputs high (LED1 lights up).

S-3: Start the Linux operating system, if the boot fails, GPIO20 outputs high (LED2 lights up).

S-4: Send control commands for conventional sensors via IIC bus and SPI bus, etc., get the status of the multiparameter sensor acquisition module consisting of one or more conventional sensors, determine if the multiparameter sensor acquisition module has been successfully connected; if the connection fails GPIO21 outputs high (LED3 lights up).

S-5: Use the USB3.0 interface to send the AT command [42] to detect the 5G module status of the Huawei MH5000-31. When the detection is successful, configure the Linux network connection and dial-up internet access to enable the blockchain smart sensor to use the 5G network for data interaction. If the connection fails, GPIO18 outputs a high level (LED4 lights up).

S-6: Register the blockchain smart sensor as a legitimate blockchain node user. Calling the blockchain client initialization program, SUBKey provides the asymmetric secret-key KSN and symmetric secret-key Key, and stores the parameters related to the blockchain client initialization process into the USBKey.

S-7: Enter the smart sensor main program. Read the data from the multiparameter sensing and acquisition module, then perform integrity and confidentiality security operations on the data and invoke the smart contract to upload the data to the blockchain.

## 4. Features and Security Analysis of 5G-BSS

### 4.1. Feature Analysis

- Decentralized Storage.

Blockchain [16,17,45,46] is a distributed ledger technology based on peer-to-peer (P2P) networks, cryptography and consensus mechanisms, evolving into a complete distributed storage system relying on logical control functions such as smart contracts. Its features include multiparty trustworthiness, nontamperability, traceability, block verifiability, etc. Data from ordinary smart sensors is mainly taken to centralized storage solutions such as local storage for sensors, local server storage and uploading to cloud storage. In the 5G-BSS designed in this paper, the multiparameter sensing collection module collects data and the core processing module (Raspberry Pi 4B) calls the Update() smart contract to upload the data to the blockchain storage. This turns the smart sensor from traditional centralized storage into a decentralized distributed storage. There are significant improvements in terms of attack resistance, collusion resistance and fault tolerance.

- Higher performance.

In terms of hardware, the RAM-based Raspberry Pi 4B microcomputer motherboard is used to exploit the core processing module of 5G-BSS. Its parameters are: Broadcom BCM2711, 1.5 GHz, 64-bit, 4 cores, ARM Cortex-A72 architecture, 1 MB shared L2 cache RAM: 4 GB LPDDR4-3200, 40pinIO. The Raspberry Pi 4B has many advantages, such as large storage capacity, fast processing speed, rich interfaces, etc. The network adapter module of 5G-BSS uses the current highest performance Huawei Baron MH5000-31 5G module, using USB3.0 to interact with the core module for data; 5G communication [11,12,13] data transmission has the advantages of large capacity and low latency. In terms of the system software, it runs on top of the Linux operating system, which can handle various tasks in multiple threads with high stability.

- Highspeed, high bandwidth communications.

In terms of communication transmission, 5G communication is used, which is a new generation of mobile communication technology with a peak rate of 20 Gbps and a transmission delay time of less than 1 ms [38,47]. Its ultra-reliable low-latency communication (uRLLC) and enhanced mobile broadband (eMBB) have features of high bandwidth and low latency [11,12,38]. The network adapter for 5G-BSS was selected from Huawei’s Baron 5000 chip industrial module, the highest performance industrial 5G communication solution in existence. Huawei MH5000-31 industrial module has many features, e.g., flexible access to different 5G standard networks, supports China’s 5G SA network construction; supports single-core full-mode, conforms to 2G/3G/4G/5G, directly synchronizes to 5G network coverage pace and protects customer equipment investment. It supports the full spectrum of NR TDD and FDD for the first time in the industry and comes with a high-performance application processor with an arithmetic power of 14,400 DMPIS. In the 5G communication test results in Section 5.3 of this paper, the Huawei MH5000-31 5G industrial module has a data upload communication bandwidth of approximately 190 Mpbs and a communication delay of approximately 1 ms in the experimental environment.

### 4.2. Security Analysis

- Data Security Analysis.

Confidentiality: The data on the blockchain is public, but this paper uses the SM4() symmetric encryption algorithm to encrypt the sensor data, so only the ciphertext data of the sensor is public on the blockchain. The purchasing user of the blockchain smart sensor has the corresponding USBKey, and only the secret key *Key* provided using the unique USBKey can decrypt the ciphertext data of the smart sensor, so that the data of the corresponding smart sensor can be viewed in real time. This ensures the confidentiality of the sensor data.

Integrity: In 5G-BSS, the SM3() hash function is used to calculate the hash value of the sensor data and the hash value can be used to verify the integrity of the data. The blockchain smart sensor calls the Updata() function to upload the processed sensor data directly to the blockchain for storage. The integrity of the data is again fully guaranteed due to the tamper-evident nature of the blockchain itself. When viewing the data, the integrity of the sensor data can be verified using the hash value.

Availability: Blockchain is a decentralized distributed storage system [15]. Decentralized storage can overcome common hazards that exist in traditional centralized storage, such as DDOS attacks, hacking of centralized storage servers, viruses, etc. Decentralized storage can avoid the following risks: hardware failure, software failure, environmental risks, human failures, natural disasters, etc. All these are to ensure the availability of blockchain smart sensor data.

- Communication transmission security analysis.

Many new technologies are used in 5G communication, such as network functions virtualization (NFV) technology, network slicing technology, etc., [48]. 5G wireless communication is characterized by high reliability and high security [49]. The Huawei MH5000-31 5G industrial module uses a dual security mechanism of microkernel plus TrustZone to make data interaction in industrial and IoT environments truly secure and reliable. Therefore, the Huawei MH5000-31 5G industrial module is chosen as the network adapter for the blockchain smart sensor designed in this paper and its data communication transmissions are highly secure. 

In the past, smart sensors mainly transmitted data to local computers or local services through certain communication methods. There are two main communication methods; one is wireless communication, such as ZigBee, Bluetooth, RFID, WiFi and infrared, and the other is wired communication, such as serial port and wired LAN. These communication processes are cumbersome and highly susceptible to security risks such as signal interference, eavesdropping and camouflage, which seriously reduce the security of communications. However, in the blockchain smart sensor designed in this paper, data communication is achieved through the network adapter (the 5G communication module) of the smart sensor; 5G-BSS invokes the data transmission smart contract module and directly uploads the sensor data to the decentralized distributed storage system; therefore, the blockchain smart sensor effectively avoids the communication security problems caused by the complex and cumbersome transmission process.

## 5. Experimental Procedure

### 5.1. Experimental Environment

**Figure 8:**
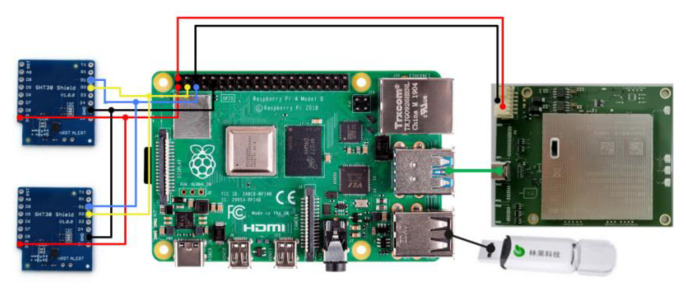
Schematic diagram of the 5G-BSS connection.

Fisco-Bcos [50] is an excellent and reliable federated blockchain and one of the top 50 blockchains in the world that has the advantages of ease of use and open source. Therefore, we choose the Fisco-Bcos as the blockchain platform for smart sensors. In this paper, a prototype experiment to implement a blockchain smart sensor based on 5G communication is implemented. The experimental platform server, is Intel(R) Xeon(R) Gold 6133CPU ×1 @ 2.5 GHz, 2 G RAM, 120 G storage, Ubuntu 18.04.4 LTS, and the local machine is Intel Core(TM) i5-10500CPU ×6 @ 3.1 GHz, 32 G RAM, 500 G storage, Windows 10. The Fisco-Bcos 2.0 federated blockchain network is deployed according to the official Fisco-Bcos documentation [50]. The smart contract deployment address for the data upload of the smart sensors in the experiment is 0xe00c3f878829d359881aa547284a32d2ea1ca3fb. The software development tool used is IntelliJ IDEA Educational Edition 2020.3.3 ×64, running in a Windows 10 environment. At the same time, the local machine Windows 10 system was installed with VMware Workstation Pro software and the virtual machine Ubuntu 18.04.4 LTS system was run. In the experiments in this paper, the multiparameter sensor acquisition module consists of 2 SHT30 conventional sensors that enable data acquisition of the physical variables of temperature and humidity of the surrounding environment. Combined with the hardware system design derived from Section 3.1, the smart sensor hardware for this experiment consists of 2 SHT30 temperature and humidity sensors, LinguoTech USBKey, Raspberry Pi 4B and the MH5000-31 5G industrial module. The 5G-BSS development board is connected schematically as shown in Figure 8.

### 5.2. Functional Verification

Firstly, for 5G communication, the hardware connection to the Raspberry Pi 4B was completed using the Huawei MH5000-31 industrial module via a USB 3.0 interface in the smart sensor Linux(OpenWrt) system to modify the Linux kernel driver and configure the USB serial port-driver related configuration items and the dial-up Internet access. This way the blockchain smart sensors use 5G communication to achieve network data transmission of the system. In the experimental platform Ubuntu 18.04.4 LTS system, the Fisco-Bcos blockchain network was set up according to the official Fisco-Bcos documentation [50]. The blockchain smart sensor calls the blockchain client program to initialize and the smart sensor node joins the Fisco-Bcos blockchain network. The transmission of transaction data is completed in the smart contract through the system network link (5G network communication). The smart contract is invoked in the blockchain smart sensor, where the data on the environmental variables of the smart sensor is automatically uploaded to the Fisco-Bcos blockchain storage. The blockchain smart sensor solution was experimentally proven to be feasible. As follows, Table 6 evaluates the validation results of the various functions that can be achieved by the blockchain smart sensor.

### 5.3. Performance Analysis

In practical application scenarios, a large amount of data on environmental variables will be collected as the use of the blockchain smart sensor device increases over time, but the federated blockchain Fisco-Bcos, as a distributed storage system, assumes a strong storage capacity, so we will not be concerned with storage overhead in this experiment. At the same time, the multiparameter sensor acquisition module consists of multiple conventional sensors, whose performance in collecting parameters of the surrounding physical variables is related to the performance parameters provided by the conventional sensors, which are not investigated here in this experiment. Therefore, in order to evaluate the performance of the blockchain smart sensors, we defined four performance metrics, namely: blockchain system throughput and blockchain system latency, blockchain client initialization time spends, data security processing module time spends and 5G communication upload rate and latency.

- Blockchain system throughput and blockchain system latency.

**Figure 9:**
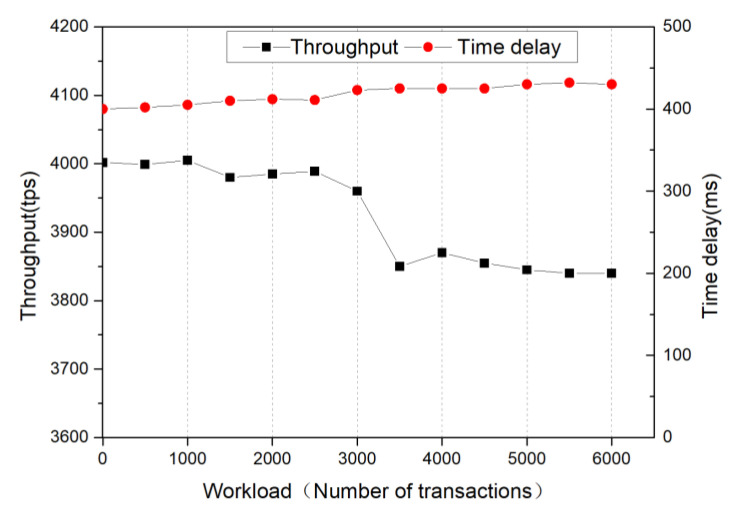
Blockchain system throughput and transaction latency test results.

Throughput is the maximum number of requests that can be processed per unit of time (seconds). Throughput is always an important metric for evaluating systems and is usually expressed in terms of reads per second, i.e., throughput = the total number of operations/total time (seconds). System latency is the time delay for a request to be responded to in the system. In the work of a blockchain smart sensor, the sensor data needs to be processed and uploaded to the blockchain distributed storage system for storage, so the throughput and the latency of the blockchain system directly affect the overall performance of the blockchain smart sensor. To test the throughput and transaction data latency of the Fisco-Bcos federation blockchain system, this test was conducted on the Fisco-Bcos federation blockchain in the experiment platform server, where min_block_generation_time [50] has defaulted to 500 ms and the test tool was JMeter. The throughput and latency test results of the blockchain system are shown in Figure 9. The throughput of the blockchain system is about 3900 tps and the maximum latency of the blockchain system is 448 ms.

- Blockchain client initialization time spends.

**Figure 10:**
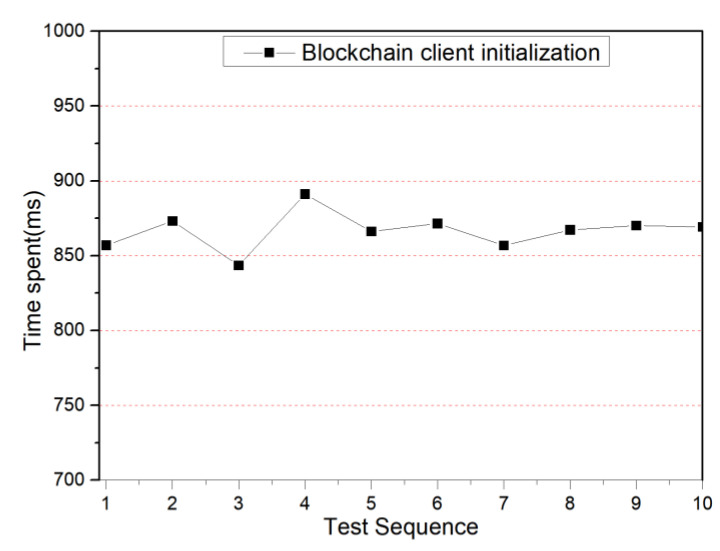
Blockchain client initialization time spends.

Each blockchain smart sensor needs to be initialized with the blockchain client program module when it is first started, and its initialization includes the registration of the blockchain client, making the blockchain smart sensor a legitimate node user of the blockchain and joining the blockchain network. In this experiment, the blockchain client initialization performance indicators are evaluated, 10 groups are tested, the average value of 10 tests is taken for each group and the test results are shown in Figure 10. As can be seen from Figure 10, the average time required for blockchain client initialization is 835 ms. The blockchain smart sensor only requires blockchain client initialization when it is first started, and its initialization time is less than 1 s, which is in line with the original design intention of this paper.

- Data security processing module time spends.

The data security processing module in the blockchain smart sensor is designed as in Section 3.2.3 of this paper, i.e., SM3() hashing operation on the sensor data to ensure data integrity and SM4() encryption operation to determine data confidentiality. This experiment is conducted for temperature and humidity collected data, where the temperature is stored in the original data format of a 38 bytes string, e.g., (S0000001 T 35.36 °C 5 July 2021 22:43:25); humidity stores a string of 39 bytes in the original data format, e.g., (S0000001 H 21.56%rh 7 July 2021 12:03:37); temperature and humidity store a string of 49 bytes in the original data format, e.g., (S0000001 T 35.36 °C H 21.56%rh 7 July 2021 12:03:37). The test SM3 time overhead is shown in Table 7 and the test SM4 time overhead is shown in Table 8.

- 5G communication upload rate and latency.

**Figure 11:**
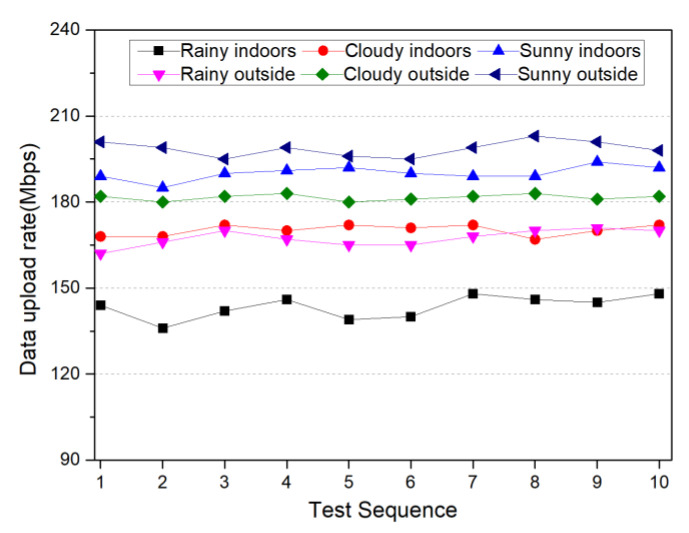
MH5000-31 5G communication industrial module data upstream transmission rate test results.

**Figure 12:**
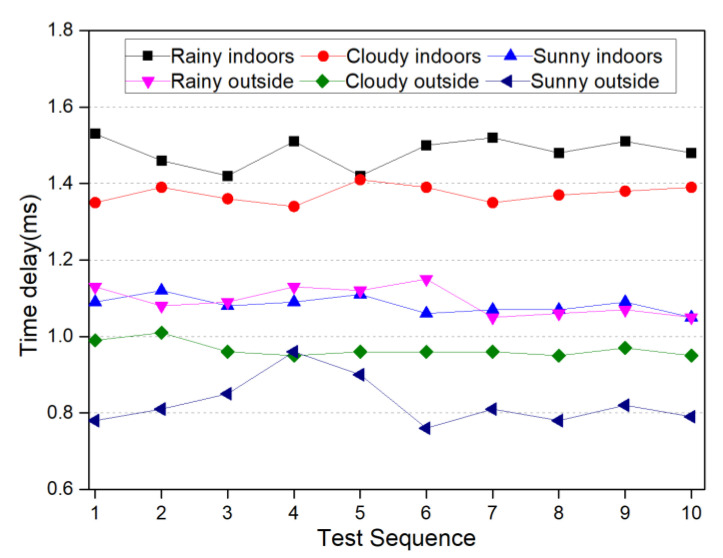
MH5000-31 5G communication industrial module network latency test results.

The performance of 5G communication is directly related to the performance of data transmission in the blockchain smart sensor. This experiment conducted data communication tests on the Huawei MH5000-31 industrial module device, which was equipped with four antennas (including one heel of the main antenna). The test software was SPEEDTEST [51], the test location was Sichuan Normal University’s Chenglong campus, where a telecom 5G base station was visually observed nearby, and the SIM card was a China Telecom 5G Platinum membership package. During the tests, we took into account the impact of buildings and weather on the quality of mobile wireless communication, so we conducted indoor and outside communication tests on rainy, cloudy and sunny days for the months of April to October. The results of the 5G communication upload rate test are shown in Figure 11 and the results of the 5G communication delay test are shown in Figure 12. Figure 11 shows that the average 5G communication upload rate is 183.57 Mbps and the lowest upload rate is about 144.62 Mbps, which means that the channel transmission delay of the smart sensor is less than 0.002 ms. Figure 12 shows that the average value of 5G communication network delay is about 1.1 ms and the maximum value is 1.5 ms.

In summary, the blockchain smart sensor (5G-BSS) designed in this paper satisfies the characteristics of rapid data processing and low data transmission latency, while also ensuring data confidentiality, security and availability.

## 6. Conclusions

There are obvious security issues with both data storage and data communication for common smart sensors. For data storage, common smart sensors mainly use local server storage or cloud server storage, both of which are vulnerable to risks such as human modification, virus invasion and database attacks. For data communication, common smart sensors mainly use Bluetooth, infrared wireless, ZigBee, UART and wired networks, etc.; these communication methods have some disadvantages such as cumbersome process stages, passing through more insecure entities and vulnerability to attacks and high latency. These two aspects of common smart sensors are highly susceptible to data leakage and data tampering. In this paper, we have designed a new smart sensor, the 5G-BSS (blockchain smart sensor based on 5G communication), where the network adapter has been chosen for 5G communication along with a client blockchain module and an encryption module. 5G-BSS has four main advantages. Firstly, the tamper-proof, decentralized and traceable trustworthiness of the blockchain is utilized to achieve the storage of sensor data in a distributed system, ensuring the security of the data. Secondly, the SM3 hash operation ensures the integrity of the sensor data and SM4 ensures the confidentiality of the sensor data. Thirdly, high-performance hardware is used, which features agile data collection, rapid data processing and fast data transmission. Fourthly, the 5G communication security protocols are perfect, with low transmission latency and high bandwidth, using 5G communication to transmit sensor data to further improve the security and real-time data. In addition, smart sensors can choose different traditional sensors to form the multi parameter sensor acquisition module according to actual application scenarios, enhancing the versatility of blockchain smart sensors.

With the booming development of the Internet of Things (IoT), the current smart sensor application scene continues to innovate. Smart sensors show the characteristics of integration, intelligence and networking, etc. As the cornerstone of IoT and artificial intelligence, smart sensors require higher security of their data, faster data transmission and stronger data processing capability. It is the trend of the time to design smart sensors with features such as high performance, security and low latency. High-performance microprocessors, 5G communications and blockchain technology empowering smart sensors are important and unstoppable developments for smart sensors.

## Figures and Tables

**Table 1:** Comparison table of the four open-source hardware parameters.

Work	5G	Blockchain	Features	Disadvantages
Paper [33]	×	√	Use only the blockchain SHA-1 algorithm	Slow and complicated data transmission, data security cannot be guaranteed
Paper [34]	×	√	Use only the temporal data database InfluxDB to store data	Slow and complicated data transmission, data security cannot be guaranteed
Paper [35]	×	√	Use BigChainDB and Distributed Ledger Technology (DLT) to be used as a blockchain-like database to store data	Slower data transfer
Paper [36,37,38]	√	×	Data transmission using 5G communication	Data storage security is not completely guaranteed
Paper [39]	√	√	5G transmission of data, using only blockchain technology to encrypt data	The security of data storage cannot be completely guaranteed
Paper [40]	√	√	A framework is proposed which uses 5G communication and blockchain technology for protecting AV systems	Complex data transmission process, data storage security is not thoroughly guaranteed

**Table 2:** Comparison table of the four open-source hardware parameters.

Model	CPU	Memory	Support Interface	Features and Application
Raspberry Pi-4B 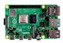	4x@ ARMCortex-A72 1.5 GHz	1 GB/2 GB/4 GB /8 GB LPDDR4	2 × USB3.0, 2 × USB2.0, 17 × GPIO, 4×I2C micro HDMI ports, etc.	Support for multiple operating systems, suitable for complex scenarios with multiple tasks, e.g., industrial internet, smart home.
Arduino R3 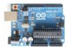	ATmega328P Clock:16 MHz	16 MB/ 32 MB	14 × GPIO, 2 × IIC, SPI, USB, UART, etc.	Low price, commonly used for creator IoT experiments, physical environment monitoring, etc.
vvBoard 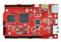	Quad-core Cortex-A53 1.5 GHz	1 GB, LPDDR3(Extended to 2G/4G)	GPIO, 1 × IIC, 1 × SPI, USB2.0, 1 × USB3.0, UART, etc.	Suitable for Python programming, mainly for the acquisition and processing of artificial intelligence data.
mirco:bitV2 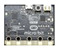	nrf52833 Clock:32, 64 MHz	Flash: 512 KB RAM: 64 KB	20 × GPIO, 1 × IIC, 1 × SPI, 1 × USB2.0, UART, etc.	Easy to use and suitable for primary and secondary school student electronic interest development and IT teaching.

**Table 3:** Comparison of 5G industrial modules.

Model	Network Format	Frequency Band	Features
MH5000-31 5G 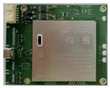	5G-NR 4G-FDD 4G-TDD 3G-WCDMA 2G-GSM	4XCA 5G:n1/41/78/79 LTE:B1/3/5/8/34/38/39/40/41 WCDMA:B1/8 GSM:900/1800	Based on domestic Baron 5000 baseband, cost effective, supports AT commands, supports Huawei’s own developed interfaces such as Modem, PCUI, Diag, GPS, GPS Control, etc.
SIM8200EA-M2 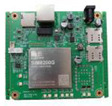	5G-NR 4G-FDD 4G-TDD 3G-WCD MA	5G-NR:n1/2/3/5/7/8/12/20/25/28/40/41/66/71/77/78 FDD:B1/2/3/4/5/7/8/12/13/14/17/18/19/20/25/26/28/29/30/66/71 TDD:B34/38/39/40/41/42/48 WCDMA:B1/2/3/4/5/8	Based on Qualcomm Snapdragon X55 baseband, expensive and supports AT commands.

**Table 4:** USBKey product comparison.

USBKey Model	Password Service	Safety Memory	Interfaces	Features
SuCIZ U1000_Smart 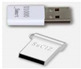	Supports multiple encryption algorithms TEA, DES, SM1, SM2, SM3, true random number operation generation, etc.	8 KB	Software: multiple language SDK development kits are available. Hardware: support for USB 2.0, etc.	On-chip software and hardware with iterative self-destruct design; driverless, hot-plugging support; Windows and Linux system support.
UltraSec USK-200 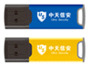	Hardware implementation of multiple packet cipher algorithms, including DES, 3DES, SM1, SSF33, SM4 and SM2 algorithms.	200 KB	Software: standard security middleware interface provided. Hardware: USB 2.0 interface.	Has a 64-bit unique hardware serial number and supports operating systems such as Windows and Linux.
LinguoTech USBKey 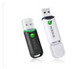	Supports SM1, SM4, DES, 3DES, RSA encryption, RSA signature verification, SM2-256 key pair generation algorithm, SM2, SM3, MD5, true random number generation algorithm, etc.	64 KB	Software: CSP interface, national secret standard interface, etc. Hardware: support for USB2.0, compatible with USB1.1, USB3.0, etc.	Rich interfaces, hot-plugging support, good support for national security algorithms. Flexible customization according to requirements, support for application layer interface calls, support for COS command layer interface calls. Supports Windows and Linux operating systems.

**Table 5:** Important symbols used in this paper and their meanings.

Symbols	Meaning
*Blockchain*	Blockchain, which is open, transparent, tamper proof and irreversible; used as a database for decentralized storage in this solution.
*SN*	Blockchain smart sensor(5G-BSS) nodes.
*SN_ID*	Blockchain smart sensors(5G-BSS) for ID.
*K_SN_*	Asymmetric secret key pairs for smart sensors(5G-BSS), including the public key *K_pubSN_* and the private key *K_priSN_*.
*Key*	Symmetric encryption secret key for Smart sensor(5G-BSS), provided by USBKey.
*Addr*	The smart sensor blockchain account address *Addr*, indicates the identity of the smart sensor administrator.
*SN_KGen()*	Interface for generating smart sensor asymmetric key pairs for *K_SN_*.
*SN_KeyGen()*	Interface for the generation of symmetric key pairs *Key* for smart sensors.
*Addr_Gen(K_pub_)*	The parameter *K_pub_* indicates the public key parameter required to generate the account address *Addr*.
*E(key,* **M** *)* *D(key,* **N** *)*	*E()* for encrypting the plaintext **M** and *D()* for decrypting the ciphertext **N**, where the *key* can be a symmetric and asymmetric key.
*USB_Write(D)*	USBKey writes data function interface, **D** is the data to be written.

**Table 6:** Functional validation of 5G-BSS.

	Blockchain Client Initialization	Data Collection	Cryptographic Operations	Hash Operations	Data Transfer
5G-BSS	√	√	√	√	√

**Table 7:** SM3 hash time overhead.

Data Format (Size/B)	Time Overhead/ms
Temperature (38)	0.159
Humidity (39)	0.161
Temperature and humidity (49)	0.173

**Table 8:** SM4 data encryption time overhead.

Data Format (Size/B)	Time Overhead/ms
Temperature (38)	0.769
Humidity (39)	0.772
Temperature and humidity (49)	0.814

## Data Availability

The data is in the paper.
